# Preeclampsia screening from the patient’s perspective

**Published:** 2016

**Authors:** B Luchian, M Neagu, L Luchian, R Vladareanu

**Affiliations:** * ”Carol Davila” University of Medicine and Pharmacy, Bucharest, Romania; Department of Obstetrics and Gynecology, “Prof. Dr. Panait Sirbu” Clinical Hospital, Bucharest, Romania; ** Carol Davila” University of Medicine and Pharmacy, Bucharest, Romania; Department of Cardiology, University Emergency Hospital Bucharest, Romania; *** ”Carol Davila” University of Medicine and Pharmacy, Bucharest, Romania; Department of Obstetrics and Gynecology, Elias Emergency Hospital Bucharest, Romania

**Keywords:** questionnaire, preeclampsia, responders, hypertension

## Abstract

Preeclampsia represents an important cause of maternal and fetal morbidity and mortality. The early identification of pregnant women at risk represents a priority in reducing preeclampsia complications.

For a better evaluation of the importance of preeclampsia screening, a questionnaire was developed.

**Methods.** The questionnaire based on 14 items was distributed online. The form was anonymously completed. All forms consisted of simple questions.

**Results.** Data from 151 completed forms were collected and analyzed. The analysis revealed the importance of arterial pressure control. 15% of the responders needed hypertensive treatment in pregnancy. They were interested in completing the preeclampsia risk test (88%).

**Conclusions.** The results suggested that preeclampsia screening and measurement of the atrial pressure may become a valuable tool for evaluating and for determining the diagnosis in question, but the possibility of emotional distress for the subjects at risk of developing the condition should be taken in consideration.

## Introduction

Hypertension in pregnancy represents an important health problem [**1**]. Preeclampsia, a pregnancy related disease is defined as a new onset hypertension after 20 weeks of pregnancy associated with proteinuria [**2**]. Despite the new discoveries in preeclampsia, it is still an important cause of maternal and fetal morbidity and mortality worldwide [**3**,**4**]. 

Many studies based on this medical condition are still being developed. The incidence of this pregnancy related condition is between 2-8% [**4**]. The high incidence is found in developing countries [**5**]. 

Even though screening tests were proposed for the early identification of this disease, there are still many steps needed to be evaluated in order to decrease the medical impact for women at risk [**6**]. The implementation of preventive strategies and diagnosis’ algorithms for preeclampsia represents a challenge for doctors and health services [**7**]. 

The majority of the studies are focused on preeclampsia screening and preventive therapies. It is very important to determine what is the point of the view of women and also how doctors inform them during the pregnancy [**8**,**9**].

## Materials and Methods

The research design was a descriptive online survey research. The population for the study consisted of 151 women. The questionnaire had 14 items. All the items were accessible and easy to understand and, most of them were based on simple yes or no questions. The data recruited from the questionnaire were anonymous. Anonymously completed forms were chosen in order to protect the rights of the participants and not to influence their answers option.

The questionnaire was sent by using email services and social online platforms. All the answers were recorded and analyzed by using Microsoft Office 360. The first items consisted of age, education, and residence (rural or urban). The next questions revealed how often they monitored the arterial blood pressure, if they had a device for monitoring and also if the doctors assessed the blood pressure during pregnancy. 

Other items of the questionnaire referred to different aspects of clinical examination, for example if the doctors in charge for the patients’ health described the risk of developing hypertension during pregnancy. Also, the study took into consideration the understanding of the importance of this ailment by the persons interviewed.

A key aspect was to appraise if existing preeclampsia risks during pregnancy would have a negative impact on the patients’ judgement. 

The last two questions addressed to the women involved in the study, referred to the existing episodes of hypertension and intake of antihypertensive treatment. 

The question regarding preeclampsia explained notes about this medical issue. In the support of the persons interviewed, the question about different therapies included the most often prescribed medicines. 

## Results

151 questionnaires were recorded and validated. All the responses were analyzed by using Microsoft Excel and graphics were made. 

**Fig. 1:**
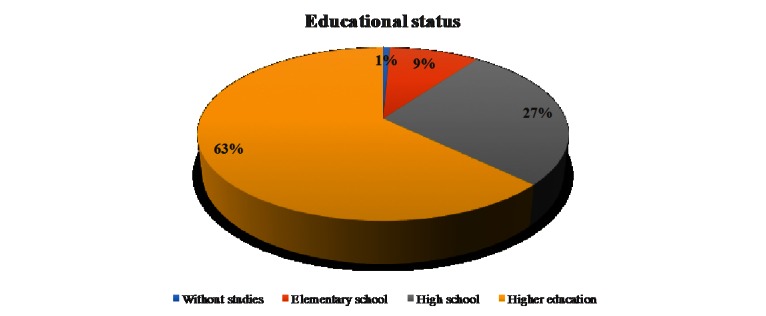
Educational status

The majority of the responders were from urban areas - 82,1%. 62,9% of the responders had university studies (**Fig. 1**).

**Fig. 2:**
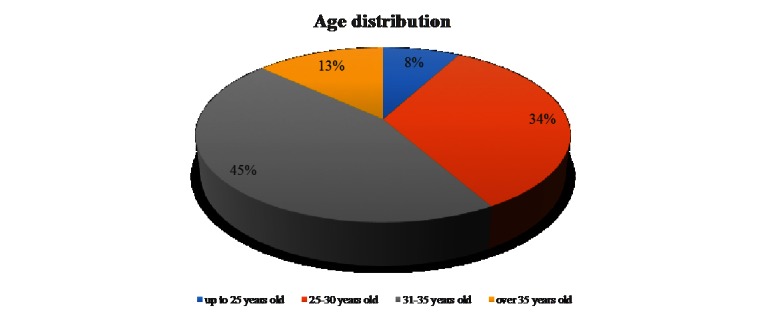
Age distribution

The age of almost 50 percent of the responders was between 31 and 35 years (**Fig. 2**).

**Fig. 3:**
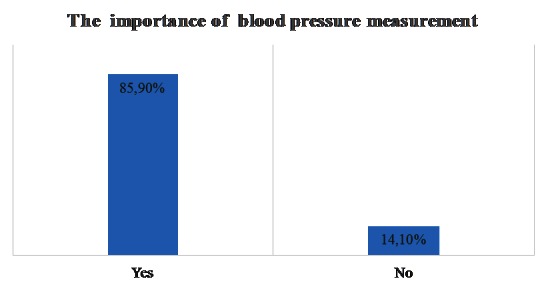
Blood pressure measurement

Most of the patients considered the blood pressure measurement important (**Fig. 3**).

Almost 70 percent of the responders had a sphygmomanometer at home. 

**Fig. 4:**
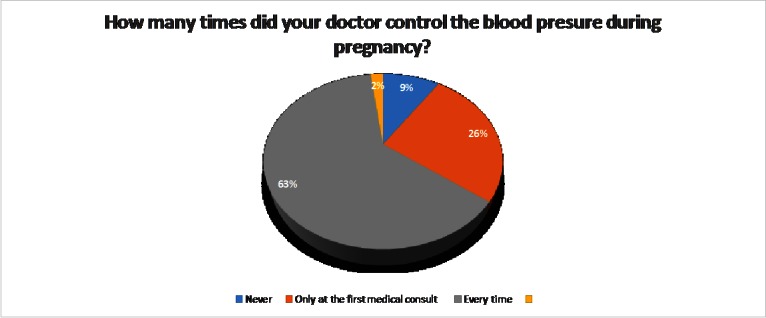
Blood pressure measurement in pregnancy

In most of the cases, the doctor measured the blood pressure during each medical consult (**Fig. 4**).

Only 60% of the questioned women were informed about hypertension and preeclampsia risks in pregnancy. 88% mentioned that they would have liked to know more about the risk. 

64% answered that knowing the risk would affect them emotionally. 

At the question regarding the personal experience of hypertensive problems during pregnancy, 75% answered positive. It was difficult to comment this answer; maybe the question should have been more concrete. The answer varied for the significance of hypertension treatment during pregnancy. 15% of the responders received therapy.

## Discussions

The findings could help in correctly managing the information about hypertension in pregnancy, especially preeclampsia. The data showed that women would like to be informed about this condition. They considered that monitoring their blood pressure during pregnancy was essential. 

As it was expected, knowing about the risk of developing preeclampsia would affect them emotionally. Being correctly informed about a medical situation in pregnancy, would affect the person’s experience. Even though the exposure to this kind of risk can be acknowledged as emotionally difficult for the women involved, learning about it could help caregivers focus on the safety and good practice care. 

The impact of preeclampsia in both mother and child wellbeing is crucial [**10**].

There are few studies about the psychological impact of preeclampsia screening test and hypertension related diseases in pregnancy [**8**,**10**]. Pregnant women deserve the right to be correctly informed. The doctors in charge play a vital role in managing these cases of women at risk of developing such a life-threatening condition.

## References

[1] East C, Conway K, Pollock W, Frawley N, Brennecke S (2011). Women’s Experiences of Preeclampsia: Australian Action on Preeclampsia Survey of Women and Their Confidants. J Pregnancy.

[2] Conde-Agudelo A, Villar J, Lindheimer M (2004). World Health Organization systematic review of screening tests for preeclampsia. Obstet Gynecol.

[3] Wright D, Akolekar R, Syngelaki A, Poon LCY, Nicolaides KH (2012). A competing risks model in early screening for preeclampsia. Fetal Diagn Ther.

[4] Rosser ML, Katz NT (2013). Preeclampsia: An Obstetrician’s Perspective. Advances in Chronic Kidney Disease.

[5] Lyell DJ, Lambert-Messerlian GM, Giudice LC (2003). Prenatal screening, epidemiology, diagnosis, and management of preeclampsia. Clin Lab Med.

[6] Park HJ, Shim SS, Cha DH (2015). Combined screening for early detection of pre-eclampsia. Int J Mol Sci.

[7] Jašović-Siveska Emiija JV (2011). Prediction of mild and severe preeclampsia with blood pressure measurements in first and second trimester of pregnancy Predykcja łagodnego i ciężkiego stanu przedrzucawkowego przy pomocy pomiarów ciśnienia tętniczego w I i II trymestrze ciąży.

[8] Harris JM, Franck L, Green B, Michie S (2014). The psychological impact of providing women with risk information for pre-eclampsia: A qualitative study. Midwifery. Elsevier.

[9] Jrgensen JM, Hedley PL, Gjerris M, Christiansen M (2014). Ethical issues related to screening for preeclampsia. Bioethics.

[10] Service NH, Pregnancy COF (2012). H Ypertension in Pregnancy Pre Eclampsia: Pre Conception Counselling for Women With a History of, or Significant Risk Factors.

